# Identification of novel *p*-cresol inhibitors that reduce *Clostridioides difficile*’s ability to compete with species of the gut microbiome

**DOI:** 10.1038/s41598-023-32656-8

**Published:** 2023-06-11

**Authors:** Mark A. Harrison, Rebecca J. Farthing, Nyasha Allen, Lucy M. Ahern, Kristian Birchall, Michael Bond, Harparkash Kaur, Brendan W. Wren, Julien R. C. Bergeron, Lisa F. Dawson

**Affiliations:** 1 Department of Infection Biology, London School of Hygiene and Tropical Medicine, Keppel Street, London, WC1E 7HT UK; 2 Randall Centre for Cell and Molecular Biophysics, King’s College London, London, WC2R 2LS UK; 3 LifeArc, Lynton House, 7-12 Tavistock Square, London, WC1H 9LT UK; 4 Department of Clinical Research, London School of Hygiene and Tropical Medicine, Keppel Street, London, WC1E 7HT UK

**Keywords:** Antimicrobials, Cellular microbiology, Microbiology, Bacteria

## Abstract

Treatment of *Clostridioides difficile* infection (CDI) is expensive and complex, with a high proportion of patients suffering infection relapse (20–35%), and some having multiple relapses. A healthy, unperturbed gut microbiome provides colonisation resistance against CDI through competition for nutrients and space. However, antibiotic consumption can disturb the gut microbiota (dysbiosis) resulting in the loss of colonisation resistance allowing *C. difficile* to colonise and establish infection. A unique feature of *C. difficile* is the production of high concentrations of the antimicrobial compound *para*-cresol, which provides the bacterium with a competitive advantage over other bacteria found in the gut. *p*-cresol is produced by the conversion of *para*-Hydroxyphenylacetic acid (*p*-HPA) by the HpdBCA enzyme complex. In this study, we have identified several promising inhibitors of HpdBCA decarboxylase, which reduce *p*-cresol production and render *C. difficile* less able to compete with a gut dwelling *Escherichia coli* strain. We demonstrate that the lead compound, 4-Hydroxyphenylacetonitrile, reduced *p*-cresol production by 99.0 ± 0.4%, whereas 4-Hydroxyphenylacetamide, a previously identified inhibitor of HpdBCA decarboxylase, only reduced *p*-cresol production by 54.9 ± 13.5%. To interpret efficacy of these first-generation inhibitors, we undertook molecular docking studies that predict the binding mode for these compounds. Notably, the predicted binding energy correlated well with the experimentally determined level of inhibition, providing a molecular basis for the differences in efficacy between the compounds. This study has identified promising *p*-cresol production inhibitors whose development could lead to beneficial therapeutics that help to restore colonisation resistance and therefore reduce the likelihood of CDI relapse.

## Introduction

*Clostridioides difficile* is the leading cause of human antibiotic associated diarrhoea, resulting in significant morbidity and mortality as well as incurring substantial economic costs^1–3^. *C. difficile* infection (CDI) usually occurs following perturbation of the gut microbiome, typically due to antibiotic treatment, allowing *C. difficile* to establish infection where the healthy microbiome would normally provide colonisation resistance^4^. CDI can range from asymptomatic infection, common in infants, to severe diarrhoea and life-threatening complications including toxic megacolon^3,5^. A major feature of CDI is the high relapse rate with up to 35% of patients suffering relapse, with those patients who suffer a single episode being at higher risk of further episodes as well^6,7^. Whilst refractory CDI can be successfully treated using faecal microbial transplant (FMT)^8,9^, FMT is expensive and there are concerns about its safety in both the short and the long term, especially regarding the introduction of unwanted pathogens^10,11^.

The interaction between *C. difficile* and the gut microbiome is crucial for the establishment and maintenance of infection, as well as for infection relapse. A key facet of *C. difficile*’s interaction with the gut microbiome is its ability to produce the phenolic antimicrobial compound *p*-cresol^12^. *C. difficile* has been shown to produce uniquely high levels of *p*-cresol, up to 25 mM^12^. Only one other species, *Blautia hydrogenotrophica*, has been shown to be able to produce *p*-cresol in the millimolar range, generating approximately 1 mM of *p*-cresol^13^, although it is unknown whether *B. hydrogenotrophica* can utilise exogenous *p*-HPA for high level *p*-cresol production in the same way that *C. difficile* is able to. Previously, it has been demonstrated that *p*-cresol selectively kills Gram-negative bacteria, which are relatively sensitive to *p*-cresol, whilst Gram-positive bacteria, such as *C. difficile*, are relatively tolerant to *p*-cresol^12^. Furthermore, microbiome analysis of an in vivo murine model of infection relapse showed a significant reduction in the abundance of Gammaproteobacteria in animals infected with wild-type *C. difficile* compared to a defined *p*-cresol deficient mutant, following vancomycin induced infection relapse^12^. In *C. difficile*, *p*-cresol is produced by the fermentation of *p*-tyrosine via the intermediate *para*-Hydroxyphenylacetic acid (*p*-HPA) which is converted to *p*-cresol via the actions of *p*-HPA decarboxylase encoded by the *hpdBCA* operon^14,15^. Each of the three genes that form the *hpdBCA* operon are essential for the function of the enzyme, with inactivation of any one of the genes leading to *C. difficile* being unable to convert *p*-HPA to *p*-cresol^15^. Recently, it was demonstrated that *C. difficile* responds to the presence of exogenous *p*-HPA via induction of the *hpdBCA* operon resulting in high level *p*-cresol production^16^. Additionally, this response and ability to produce *p*-cresol is universal for *C. difficile*, with the HpdBCA pathway conserved in all five lineages suggesting that targeting this virulence factor would be widely effective against CDI^17^.

The HpdBCA decarboxylase complex is a glycyl radical enzyme, which catalyses the decarboxylation of *p*-HPA and 3,4-Dihydroxyphenylacetate^14^. The structure of the HpdBCA decarboxylase in *C. difficile* has not yet been determined. However, in the related *Clostridium scatologenes* species the structure of the homologous 4-Hydroxyphenylacetate decarboxylase (4-HPAD_Cs_) complex has been solved^18^. In *C. scatologenes*, 4-HPAD_Cs_ has a canonical glycyl radical enzyme (GRE) topology made up of one catalytic subunit (ß) and one γ-subunit, which in turn coordinates two iron-sulphur clusters^18^. Yet, it is distinguished from most other GREs as its concomitant activating enzyme (AE), HpdA, initiates oligomerisation^19,20^. Whilst archetypal GREs are homodimeric, HpdBC is an octamer, consisting of four copies of the heterodimer (ßγ)^20^. Previously, it was demonstrated by Selmer et al. that the *C. difficile* HpdBCA decarboxylase could be inhibited by the substrate analogues *p*-Hydroxyphenylacetamide and *p*-Hydroxymandelate when tested in cell free extracts generated from cells expressing the decarboxylase^14^. However, the efficacy of these compounds in inhibiting *p*-cresol production with *C. difficile* cells or reducing *C. difficile*’s ability to compete with other species of the gut microbiome was not determined.

In this work we have identified several promising inhibitors of *p*-cresol production, with the lead compound, 4-Hydroxyphenylacetonitrile, significantly reducing *C. difficile*’s ability to compete with a gut dwelling *Escherichia coli* strain in competition-index assays, through a reduction in *p*-cresol production. In silico molecular docking experiments provided structural insight into how the inhibitors interact with the HpdBCA decarboxylase and allowed us to propose a molecular basis for the range of inhibitions obtained. This work paves the way for structure activity relationship (SAR) modification of the inhibitors to refine these HpdBCA decarboxylase inhibitors for use as a potential treatment for *C. difficile*. Given that *C. difficile* is one of only a few gut bacteria that produce *p*-cresol, therapeutics targeted against *p*-cresol could be highly specific to *C. difficile* thus having little impact on the microbiome and therefore they could be a valuable tool in the ongoing battle against CDI.

## Methods

### Compound identification

The 4-HPAD_Cs_ structure (PDB ID: 2YAJ) shows the substrate bound in the active site, tightly enclosed in the protein core. The apo structure (PDB ID: 2Y8N) is similar; both lack a substrate sized channel between the active site and exterior of the enzyme, meaning that there must be some degree of conformational plasticity to allow passage. The few known inhibitors^14^ provide limited information on the tolerance of groups in the binding pocket. We sought to explore a wider range of substrate analogues to probe the plasticity of the pocket, discover improved inhibitors and provide data to inform inhibitor design in future. Using a combination of substructure and similarity searching of catalogues of commercially available compounds, we identified 29 close analogues (supplementary Table 1), three of which were known substrates (4-Hydroxy-3-methoxyphenylacetic acid, 4-aminophenylacetic acid and 4-Methoxyphenylacetic acid) and three were known inhibitors (4-Hydroxyphenylacetamide, 3,4-Dihydroxyphenylacetic acid and 2-Hydroxy-2-(4-hydroxyphenyl)acetic acid). Eight of these compounds were acquired for testing.

Each of the test compounds was assigned a number as follows: compound 1: 3-(4-Hydroxyphenyl)propionic acid, compound 2: Methyl 4-Hydroxyphenylacetate, compound 3: 2-(4-Hydroxyphenyl)ethanol, compound 6: 4-Hydroxyphenylacetamide, compound 8: 4-Hydroxyphenylacetonitrile, compound 9: 3,4-Dihydroxyphenylacetic acid, compound 17: 4- Hydroxybenzoic acid, compound 19: DL-4-Hydroxy-3-methoxymandelic acid.

### Growth conditions and strains used in study

*C. difficile* 630∆*erm* (derived from the 630 strain—ribotype 012, clinical isolate Zurich, Switzerland 1982)^21^ and *E. coli* (isolated from a gut soup model of CDI by Dr Simon Baines) were grown on agar plates with Brain Heart Infusion (Oxoid) supplemented with 5 g/l yeast extract (Sigma) and 0.05% L-cysteine hydrochloride (BHIS). All strains were grown on a shaker at 50 rpm in anaerobic conditions in a Modular Atmosphere Control System 500 (Don Whitley Scientific) at 37 °C. Media underwent a minimum of a 4 h pre-equilibration in anaerobic conditions prior to inoculation. Throughout the described assays *p*-HPA was used at a concentration of 1.5 mg/ml (6.6 mM) with the test compounds under evaluation matched to a concentration of 6.6 mM. Where necessary compounds were dissolved in DMSO such that the final concentration of DMSO in the growth media was 1%. Where DMSO was not necessary for dissolution, it was added to the media (to a final concentration of 1%) to ensure that the conditions the compounds were tested under were identical.

### Growth curves

Each strain was grown overnight in defined minimal media (MM) as per Cartman et al.^22^ prior to back dilution to an OD_590 nm_ of 0.5. 1 ml of back diluted culture was added to 10 ml of the test conditions in a 50 cm^3^ tissue culture flask, i.e. MM, MM + 6.6 mM *p*-HPA, MM + 6.6 mM test compound, and MM + 6.6 mM *p*-HPA + 6.6 mM test compound. OD_590 nm_ was taken every hour for 8 h and all growth curves were carried out in a minimum of biological triplicate. ANOVA analysis was carried out using GraphPad Prism 8 software to determine whether there were any significant differences between growth curves where test compounds were present compared to where they were absent.

### Preparation of samples of 630∆*erm* for *p*-cresol production analysis

*C. difficile* strain 630∆*erm* was grown overnight in MM prior to back dilution to OD_590 nm_ of 0.5. 1 ml of back diluted culture was added to 10 ml of the test conditions in a 50 cm^3^ tissue culture flask, i.e. MM + 6.6 mM *p*-HPA or MM + 6.6 mM *p*-HPA + 6.6 mM putative inhibitor. After 8 h the OD_590 nm_ was measured and 1 ml of the culture was removed, filter sterilised using 0.22 µm filters and immediately stored at – 80 °C. High performance liquid chromatography with diode array detection (HPLC–DAD) analyses to measure *p*-HPA and *p*-cresol concentrations was carried out as described below. Three biological replicates of samples were prepared for and underwent analysis. *p*-cresol production was normalised to the OD_590 nm_ to account for any differences in growth. Analysis was carried out by linear regression using StataSE 17 software to determine if any significant differences were found in *p*-cresol production when the test compounds were present compared to growth in *p*-HPA alone. *p* < 0.05 was considered statistically significant.

### Competition-index experiments

Monocultures of both *C. difficile* 630∆*erm* and *E. coli* were grown overnight in MM. A monoculture of *C. difficile* 630∆*erm* was diluted to an OD_590 nm_ of 0.2, with 18 µl of the dilution being added to 1.8 ml of the test conditions (giving a start OD_590 nm_ of 0.002): MM, MM + 6.6 mM *p*-HPA, MM + 6.6 mM test compound, and MM + 6.6 mM *p*-HPA + 6.6 mM test compound, in a 24-well plate and grown for 8 h at which point the overnight monoculture of *E. coli* was back-diluted to an OD_590 nm_ of 0.2 and 18 µl was inoculated into the *C. difficile* wells to give the co-culture. The co-culture was incubated for 14 h. The proportion of *C. difficile* and *E. coli* in the co-culture was determined by colony-forming units per millilitre (CFU/ml) assay with co-cultures plated on to BHIS plates in duplicate with selective media for each species. 1 ml of the co-culture was removed from the wells and underwent serial ten-fold dilution in phosphate buffered saline (PBS) with dilutions to 10^−6^. CFU/ml were counted the following day after 16 h incubation under anaerobic conditions on selective BHIS agar. The selective media for *C. difficile* was cycloserine (250 mg/l) and cefoxitin (8 mg/l) and *E. coli* was selected with vancomycin (4 mg/l). An aliquot of the undiluted culture was filter sterilised (0.22 µm filter) and were immediately frozen at – 80 °C for determination of the concentrations of *p*-HPA and *p*-cresol using HPLC–DAD analyses as described below. Regression analysis was carried out using StataSE 17 to determine if there were: (1) any significant differences in the proportion of the co-culture formed by *C. difficile* 630Δ*erm* between growth conditions, (2) any significant differences in *p*-cresol production between growth conditions. *p* < 0.05 was considered statistically significant.

### HPLC–DAD analyses

As described above, samples undergoing HPLC–DAD analysis were taken from (i) *C. difficile* 630Δ*erm* grown on its own and (ii) competition-index assays with *E. coli* and *C. difficile*. The filter-sterilized samples were transferred to HPLC vials and analysed immediately by using the Ultimate 3000 system (Thermo Fisher Scientific). Separations were achieved utilizing an Acclaim™ 120 C_18_, 5 μm 120 Å (4.6 × 150 mm) column (Thermo Fisher Scientific), with the mobile phase consisting of ammonium formate (10 mM, pH 2.7) and menthol (v/v; 50:50) at a flow rate of 1400 μl /min. *p-*HPA and *p*-cresol were detected by the detector (DAD 3000) set at 280 nm. Peak identity was confirmed by measuring the retention time of commercially available *p-*HPA and *p*-cresol, and determination of absorbance spectra was performed using the DAD. A calibration curve of each compound was generated by Chromeleon (Dionex software) using known amounts of the reference standards (0–5 mg/ml) dissolved in media and injected onto the column, and the amount of *p-*HPA and *p*-cresol in the samples was determined. Samples from three independent biological replicates were analysed compared to media controls and standard curves. The limit of detection for *p*-HPA and *p*-cresol were 0.001 and 0.0005 mg/ml, respectively.

### Homology model generation and preparation

HpdBC is a functional hetero-octamer with one binding site per heterodimer, in the core of the beta subunit^18^. Thus, only the catalytic subunit was required for subsequent molecular docking calculations. The full-length *C. difficile* HpdB protein sequence was obtained from the UniProt Consortium (https://www.uniprot.org/) (accession ID: Q84F16)^23^ and a model was generated using SWISS-MODEL (https://swissmodel.expasy.org/)^24^, using the crystal structure of HpdB (PDB ID: 2Y8NA, 1.75 Å resolution) from *C. scatologenes*^18^ as a template. The *C. difficile* and *C.* *scatologenes* protein sequences have a shared identity of 58.7% across their HpdB subunits, with conservation of all active site residues, as identified by Martins et al.^25^.

### Preparation of receptor and ligand for docking simulations

The substrate-bound *C. scatologenes* HpdB structure (PDB ID: 2YAJA, 1.8 Å resolution) was used as a positive control for the docking protocol. For both the *C. scatologenes* HpdB crystal structure and the *C. difficile* HpdB homology model, the monomer was prepared and optimised using the default parameters in the Molecular Modelling Toolkit^25^ integrated within the UCSF Chimera package (1.16 version), during which water molecules were removed, hydrogens added and partial charges assigned.

The 2D structures of the eight compounds tested were acquired from the PubChem database^26^. The native substrate, *p*-HPA, was derived directly from the *C. scatologenes* HpdB crystal structure (PDB ID: 2YAJ)^18^. All ligands were refined for docking using Chimera^27^ as described above.

### Molecular docking

The *C. scatologenes* HpdB crystal structure was used to define the active site grid coordinates in PyMOL (2.5 version) (The PyMOL Molecular Graphics System, Version 2.0 Schrödinger, LLC), by finding the centre-of-mass of the binding site residues as detailed by Martins et al.^18^. A PDBQT file for molecular docking simulations was subsequently generated using Autodock Tools (ADT)^28^ using X, Y and Z coordinates of 22.598, 73.951 and 46.966, respectively, and a grid box radius of 20 Å^3^. Autodock Vina 1.1.2 was used to perform the docking experiments^29^. The outputs were visualised in ChimeraX to identify intermolecular interactions and potential atomic clashes.

## Results

### Test compounds have modest or no effect on *C. difficile* or *E. coli* growth in monoculture

In the initial stages of screening, growth curve analyses were carried out to determine whether any of the putative inhibitors had significant effects on the growth of either *C. difficile* 630Δ*erm* or *E. coli*, as an important factor in the development of anti-*C. difficile* therapies is the need for them to be microbiome sparing. *C. difficile strain* 630Δ*erm* is from clade 1 and is used as a laboratory reference strain^21^. The *E. coli* strain used in this study was selected as it is a commensal strain from the “gut soup” model of infection which utilises faecal matter from healthy volunteers^12^.

**Figure 1:**
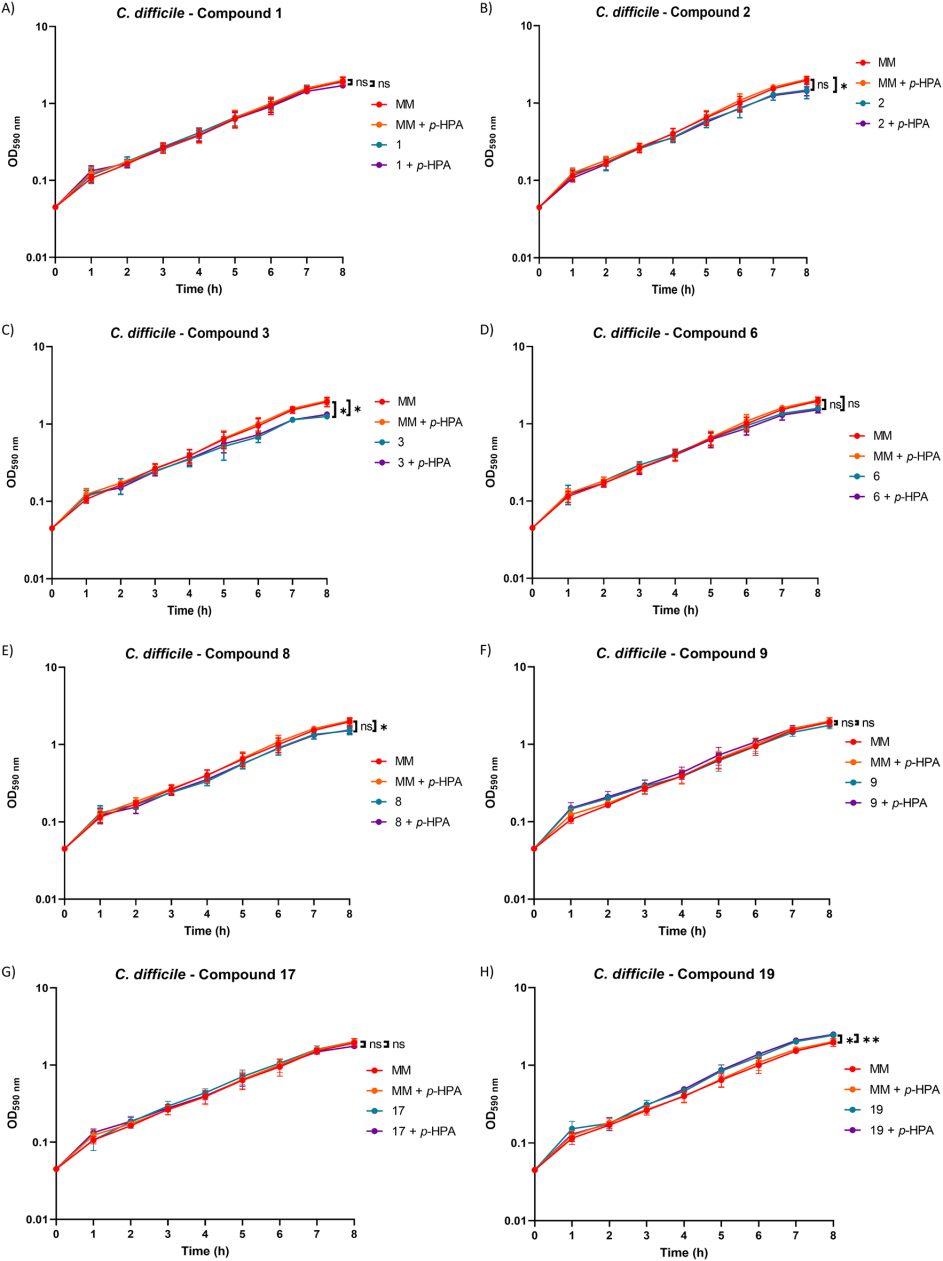
Growth of *C. difficile* 630Δ*erm* in the presence of test compounds. Growth analysis of 630Δ*erm* over the course of eight hours was undertaken in minimal media (MM) alone, MM with 1.5 mg/ml *p*-HPA (6.6 mM), MM with 6.6 mM of a test compound, or MM with both *p*-HPA and a test compound at 6.6 mM. Data represents the mean and standard deviation of three independent replicates. Statistical analysis was carried out by ANOVA and significant differences are indicated: *p < 0.05; **p < 0.01. All graphs were generated in GraphPad Prism 8.

**Figure 2:**
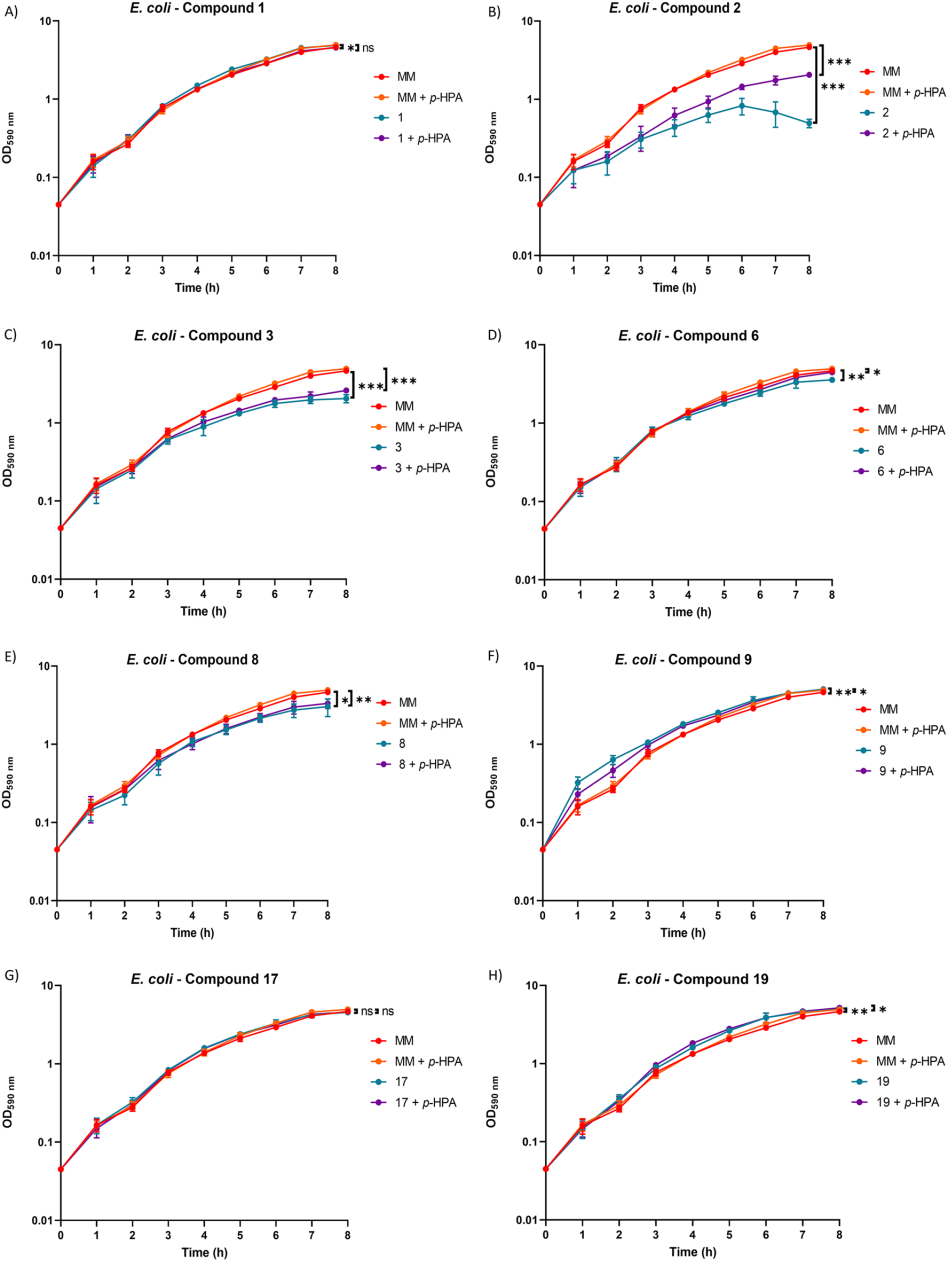
Growth of gut dwelling *E. coli* in the presence of test compounds. Growth analysis of *E. coli* over the course of eight hours was undertaken in minimal media (MM) alone, MM with 1.5 mg/ml *p*-HPA (6.6 mM), MM with 6.6 mM of a test compound, or MM with both *p*-HPA and a test compound at 6.6 mM. Data represents the mean and standard deviation of three independent replicates. Statistical analysis was carried out by ANOVA and significant differences are indicated: *p < 0.05; **p < 0.01. All graphs were generated in GraphPad Prism 8.

For 630Δ*erm*, no significant growth defects were found in any conditions with compounds 1, 6, 9 and 17, and, interestingly, we observed that compound 19 led to significantly higher growth compared to growth in MM alone (Fig. 1). Compounds 2 and 8 were found to cause a significant growth defect when *p*-HPA was present in addition to the test compounds but not without *p*-HPA also being present. Compound 3 was found to cause a significant growth defect regardless of the presence of *p*-HPA (Fig. 1). In *E. coli* only compound 17 was found not to affect growth under any condition tested (Fig. 2). In contrast, compound 1 was found to enhance growth when added on its own but not when *p*-HPA was also present whilst compounds 9 and 19 were found to enhance growth regardless of *p*-HPA being present or not (Fig. 2). Compounds 2, 3, 6 and 8 were all found to cause significant growth defects both in the presence and absence of *p*-HPA (Fig. 2).

### Quantification of *p*-cresol production inhibition by test compounds

**Figure 3:**
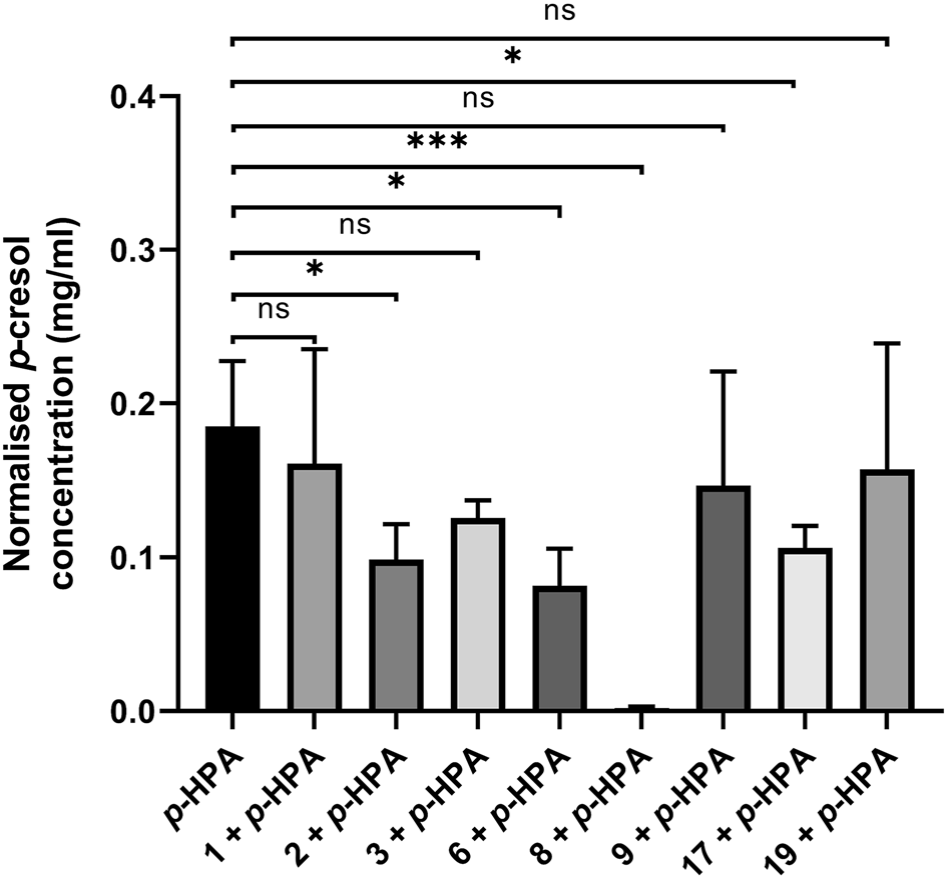
Quantification of *C. difficile* 630Δ*erm p*-cresol production in the presence of test compounds. 630Δ*erm* was grown in the presence of 1.5 mg/ml *p*-HPA, or with the addition of a matched concentration (6.6 mM) of the test compounds for 8 h. *p*-cresol concentration was normalised to the OD_590 nm_ of the culture at time the sample was taken. Statistical analysis to determine significant differences in normalised *p*-cresol concentration was carried out by regression using StataSE 17. Data represents three independent replicates, error bars represent standard deviation and significant differences are indicated: *p < 0.05; ***p < 0.001. Graph generated in GraphPad Prism 8.

To determine whether the compounds had a significant effect on *p*-cresol production, 630Δ*erm* was cultured in the presence of *p*-HPA and an equal concentration of each compound for 8 h. Quantification of *p*-cresol was carried out using HPLC which showed that under these conditions significant reductions in *p*-cresol concentration, after normalisation to OD_590 nm_, were identified with compounds 2, 6, 8 and 17 (Fig. 3). The largest decrease found was with compound 8 which reduced *p*-cresol production by 99.0 ± 0.4%, this was followed by reductions of 54.9 ± 13.5%, 46.6 ± 3.9% and 41.6 ± 7.3% for compounds 6, 2 and 17 respectively (Fig. 3). In addition, compound 3 was found to give a *p*-value approaching significance (*p* = 0.0528) with *p*-cresol production decreased by 29.1 ± 16.3% (Fig. 3). No significant changes in *p*-cresol production were identified with compounds 1, 9 and 19, suggesting they do not inhibit the HpdBCA decarboxylase (Fig. 3).

### Competition-index assays show decreased *C. difficile* competitiveness versus *E. coli* in the presence of potential HpdBCA decarboxylase inhibitors

**Figure 4:**
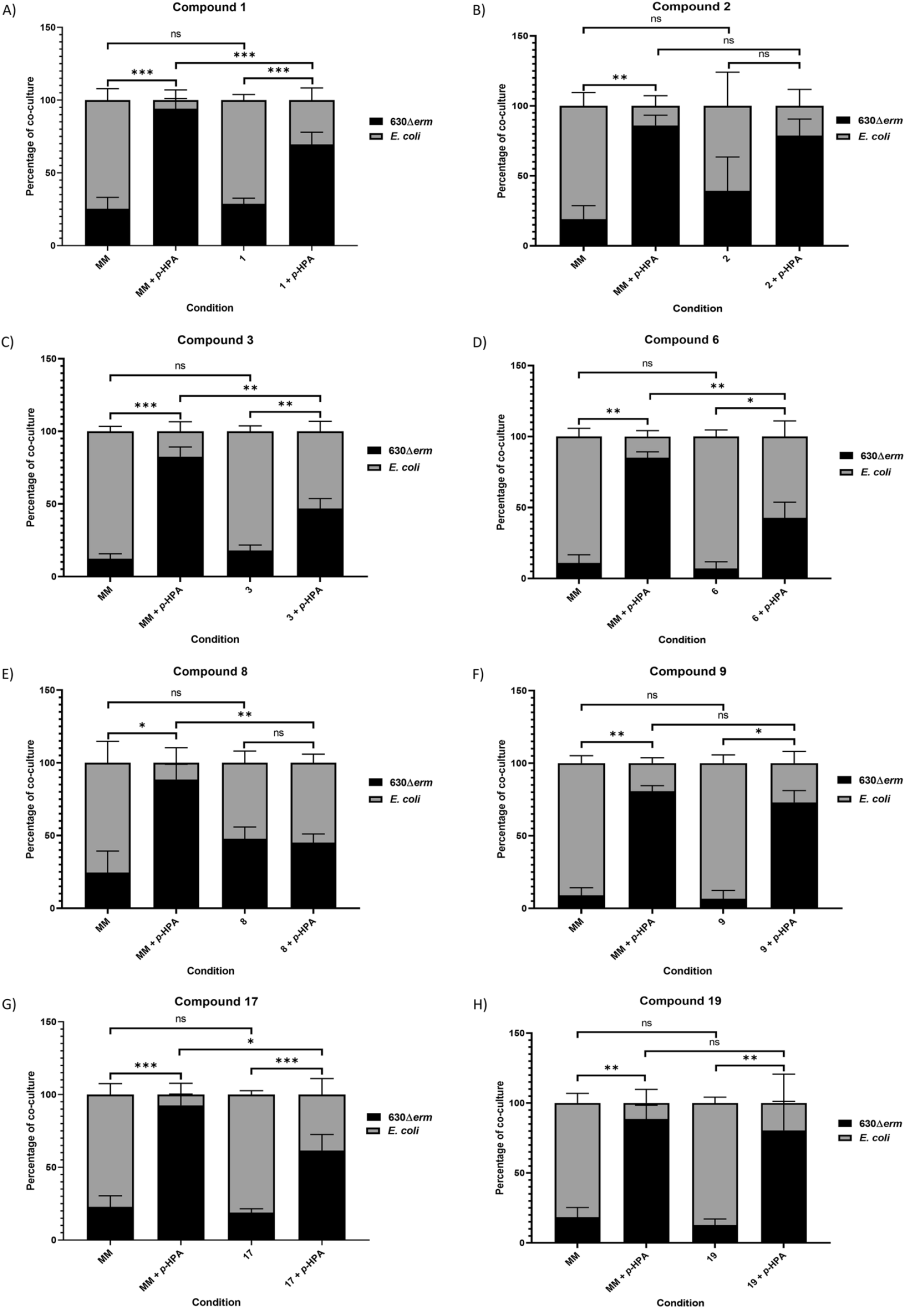
Competition-index assays of *C. difficile* 630Δ*erm* with *E. coli* in the presence of *p*-HPA and test compounds. The effect of *p*-HPA and the test compounds on 630Δ*erm*’s ability to compete with *E. coli* were determined by competition-index assays. The percentage of the culture of each species was determined by CFU/ml carried out with selective plating. Data represents the means and standard deviations of a minimum of three independent replicates. Regression analysis was carried out using StataSE 17 to determine significant differences in the proportion of 630Δ*erm*, significant differences are indicated by: *p < 0.05; **p < 0.01; ***p < 0.001. All graphs were generated in GraphPad Prism 8.

Previously, competition-index assays were used to determine that mutation of the *hpdBCA* operon, which results in an inability to produce *p*-cresol, results in *C. difficile* being less able to compete with bacteria of the gut microbiome^12^. Here, we used a similar competition-index method to screen the putative inhibitors of *p*-cresol production. In agreement with previous results, *p*-HPA supplementation into the growth media significantly increased the proportion of *C. difficile* 630∆*erm*, compared to *E. coli* in the competition-index assays*.* In the absence of *p*-HPA, *C. difficile* 630∆*erm* comprised 18.1 ± 9.1%, whereas in the presence of 1.5 mg/ml *p*-HPA the average proportion was increased to 87.5 ± 7.6% (Fig. 4). When media was supplemented with *p*-HPA as well as one of the test compounds this provided the conditions to determine if *C. difficile* was less able to compete with *E. coli* as a result of these putative *p*-cresol production inhibitors. In line with the reductions in *p*-cresol production identified by HPLC–DAD, we found that compounds 6, 8 and 17 significantly reduced the proportion of *C. difficile* 630∆*erm* in the culture compared to growth in the presence of *p*-HPA alone (Fig. 4D,E,G). Furthermore, compound 3 was also found to significantly decrease the relative proportion of *C. difficile* to *E. coli* in this assay (Fig. 4C). Surprisingly, compound 1, which did not have a significant effect on *p*-cresol production (Fig. 3) was found to significantly reduce *C. difficile* in the competition-index assays (Fig. 4A). The largest decreases in the proportion of 630∆*erm* were found with compounds 3, 6 and 8, which all show decreases of greater than 35% of *C. difficile* relative to *E. coli* in the competition-index assay (Fig. 4C,D,E). The drop in relative proportion of *C. difficile* 630∆*erm* with compounds 1 and 17 was more modest at 24.6 and 31.0% respectively (Fig. 4A,G). Conversely, compound 2, which showed a significant reduction in *p*-cresol production by HPLC–DAD (see above), did not have a significant effect in the competition-index assays (Fig. 4B). In line with the HPLC–DAD results we also observed that compounds 9 and 19 did not have any significant effects on the proportions of *C. difficile* in the competition-index, again suggesting that these compounds did not inhibit *p*-cresol production (Fig. 4F,H).

**Figure 5:**
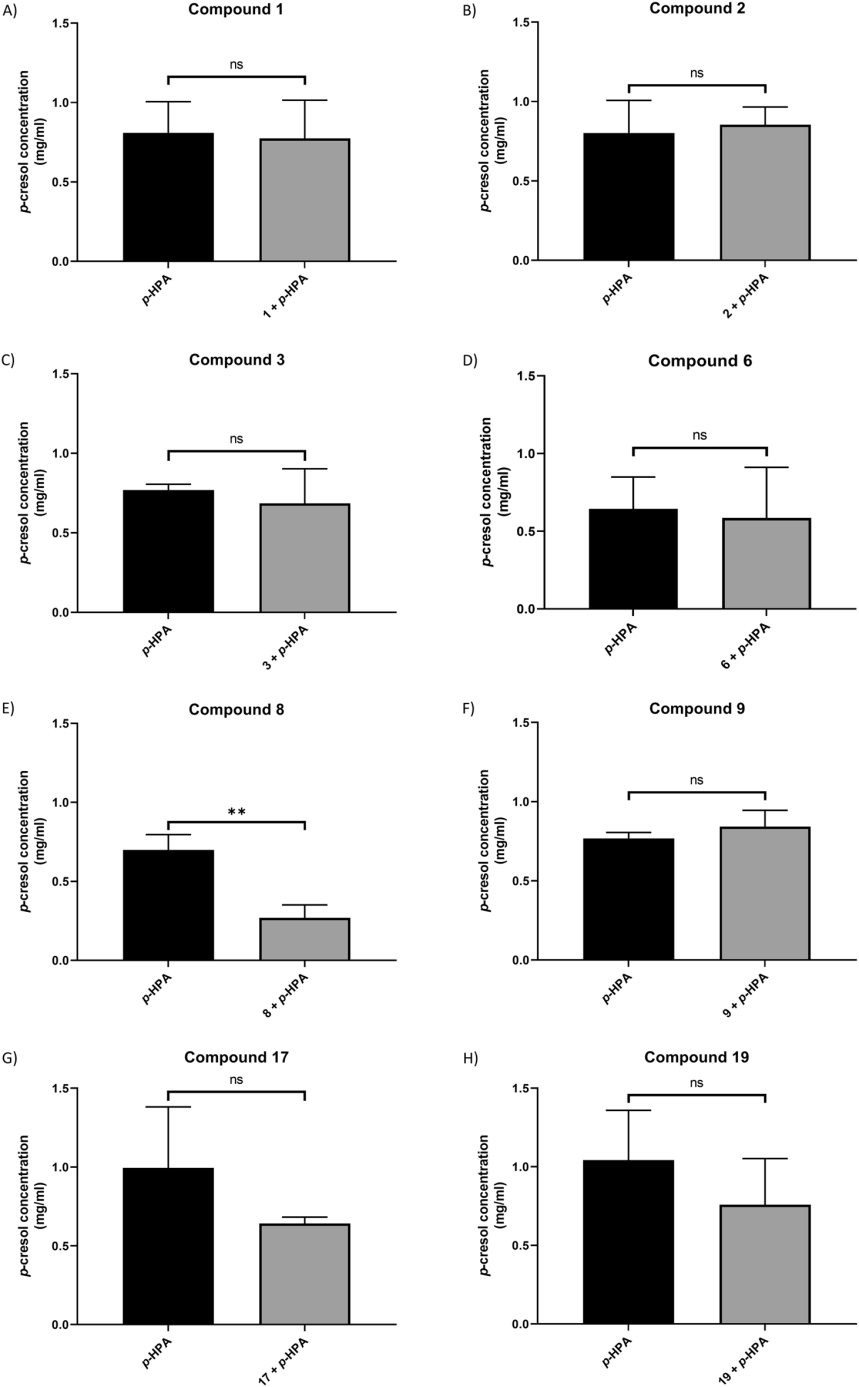
*p*-cresol concentration following competition-index assays of *C. difficile* 630Δ*erm* and *E. coli*. 630Δ*erm* and *E. coli* underwent competition-index assays, the bacteria were co-cultured in the presence of *p*-HPA with and without the test compounds for 14 h to determine how these compounds affected *C. difficile*’s ability to compete with *E. coli*. Where samples were available at the end of the co-culture assays HPLC analysis was performed to determine *p*-cresol concentration. Data represents the means and standard deviations of a minimum of two independent replicates. Regression analysis was carried out using StataSE 17 to determine significant differences in the *p*-cresol concentration, significant differences are indicated by: **p < 0.01. All graphs were generated in GraphPad Prism 8.

As outlined, both CFU quantification and HPLC–DAD analysis were performed in parallel to determine *C. difficile* relative abundance and *p*-cresol concentration respectively. The HPLC–DAD analysis revealed that the only significant reduction in *p*-cresol production compared to growth in *p*-HPA alone was observed with compound 8 (reduction of 61.5 ± 8.4% identified (Fig. 5E)). No other compounds were identified as significantly reducing *p*-cresol production in the competition-index assays (Fig. 5).

### In silico docking of the test compound inhibitors

The data reported above suggest that several of the test compounds inhibit HpdBCA’s decarboxylation activity. We therefore sought to characterize the molecular details of their interaction with the enzyme complex using molecular docking experiments. To this end, we generated a homology model of the *C. difficile* HpdB protein*,* using the crystal structure of the *C.* *scatologenes* orthologue (PDB ID: 2Y8NA)^18^ as a template. As expected, the *C. difficile* model predicts a canonical GRE topology comprising a central antiparallel β-barrel surrounded by α-helices^1,4^ and the root-mean-square deviation (RMSD) between the *C.* *scatologenes* HdpB crystal structure and its *C. difficile* homology model is 0.201 Å (Supplementary Fig. 1). Two finger-like loops protrude from the barrel, each harbouring one member of the catalytic dyad: the radical storage residue Gly877, and Cys507^1,4^.

As a positive control, we docked the donor substrate, *p*-HPA, into the binding pocket of the *C. scatologenes* HpdB, for which a crystal structure had been published previously (PDB ID: 2YAJA)^18^. Three ligand conformations were generated, and the lowest free energy prediction was selected as the preferential orientation. As shown in Supplementary Fig. 2, the position of the ligand obtained from the docking simulation is nearly identical to that of the experimentally determined structure, confirming the suitability of our docking procedure. We also performed the same procedure using the *C. difficile* HpdB homology model. As shown in Supplementary Fig. 2, the substrate docks in a very similar position to that described above, and notably forms the same hydrogen bond interactions with the active site residues of the *C. difficile* HpdB model as with the *C. scatologenes* crystal structure. The binding energies of the best-docked conformations for the *C. scatologenes* HpdB crystal structure and the *C. difficile* HpdB homology model were − 6.7 kcal/mol and − 6 kcal/mol, respectively.

**Figure 6:**
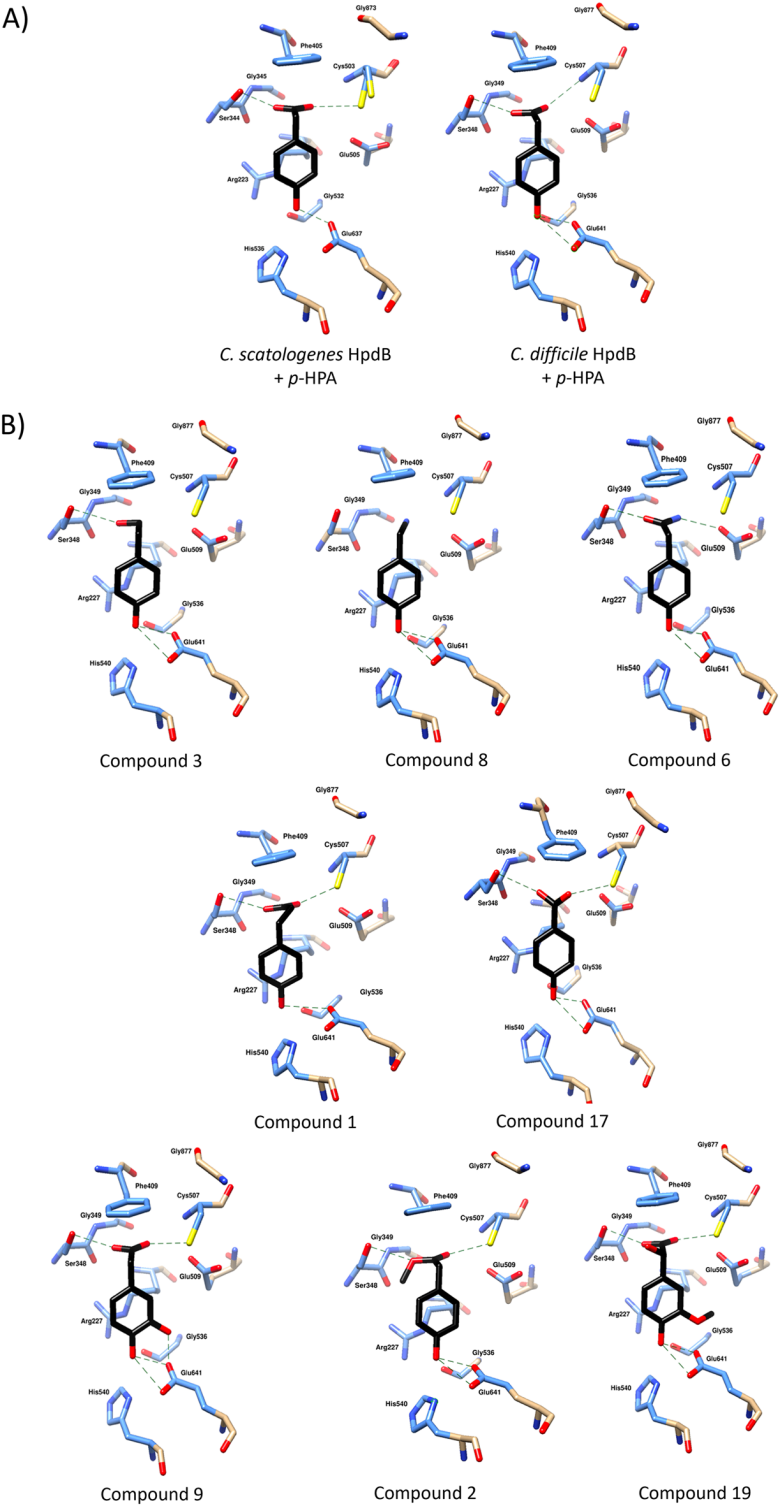
Orientation of all compounds docked in active site of HpdB. Panel (**A**) shows native substrate (*p*-HPA) self-docked in active site of *C. scatologenes* HpdB crystal structure (left) and *C. difficile* homology model (right). Panel (**B**) shows *p*-HPA docked in *C. difficile* homology model active site ordered by their predicted binding energy from lowest to highest. Active site residues only are shown as sticks. All atoms within 5 Å of ligand are coloured pale blue, atoms further away are in beige. Dashed lines show hydrogen bonds between compound and active site residues. Image generated in UCSF ChimeraX.

We next attempted to dock all the experimentally tested compounds described above, into the *C. difficile* HpdB homology model. All compounds successfully docked into the active site, and exhibited a clear top-ranked conformation, with the same binding mode as the substrate *p*-HPA (Fig. 6). The binding affinities for each ligand ranged from − 5.7 to − 2.0 kcal/mol (Table 1). Two of the inhibitors identified in this study, compounds 3 and 8, exhibit higher docking scores than compound 6, which had previously been shown to be a potent inhibitor of HpdB^14^. The binding energies of all three of these compounds are − 5.7, − 5.4 and − 5.1 kcal/mol, respectively, which are comparable with that of the value observed for the substrate docking (Table 1), suggesting a similar binding affinity. Similarly, compound 1 has the same docking energy as the positive control compound 6, which may support the observed significant *C. difficile* reduction in the competition-index assays although not the results from the HPLC–DAD analysis of *p*-cresol production. Compounds 9, 17, 2 and 19 have significantly higher binding energies, which suggests that they likely have a lower binding affinity. These results are largely in line with the above experimental characterization of the reduced inhibitory activity for these compounds with the exception of compound 17.

**Table 1:** Energies for binding of test compounds to the *C. difficile* HpdB model, estimated by AutoDock Vina^29^.

Ligand bound to *C. difficile* model	Binding energy (kcal/mol)
*p*-HPA	− 6.0
Compound 3	− 5.7
Compound 8	− 5.4
Compound 6	− 5.1
Compound 1	− 5.1
Compound 9	− 4.7
Compound 17	− 4.6
Compound 2	− 3.8
Compound 19	− 2.0

Predicted docking energies are displayed for each compound in ascending order, as compared to that of the native substrate (*p*-HPA).

Our docking protocol provides insight into the interactions between the compounds and HpdB that are important for the inhibition of *p*-cresol production. Specifically, the previously determined crystal structure had shown that *p*-HPA is entirely buried in the active site and interacts with HpdB through an extensive hydrogen bonding network. The carboxyl group of *p*-HPA is in close proximity to the active site cysteine, which facilitates the Kolbe-type decarboxylation catalysis^18^. In agreement with Martins et al.^18^, our docking results indicate that *p*-HPA binds to the *C. difficile* HpdB via its carboxyl group’s interaction with Ser348, Cys507, and Glu509, while its hydroxyl group is coordinated in a downward position by His540 and Glu641. The critical roles of these residues in the production of *p*-cresol have previously been described, and notably, Cys507 was shown to be activated by the protein-bound glycyl radical, Gly877, via the removal of a hydrogen atom^19,30,31^. The resulting thiyl radical can then attack the substrate by removing an electron and a third key active site residue, Glu641, abstracts a proton to create the enzyme intermediate^5^. Glu509 is responsible for later donating a proton to Cys507, allowing the substrate to claim a hydrogen atom from this cysteine to complete the formation of *p*-cresol^5^.

**Figure 7:**
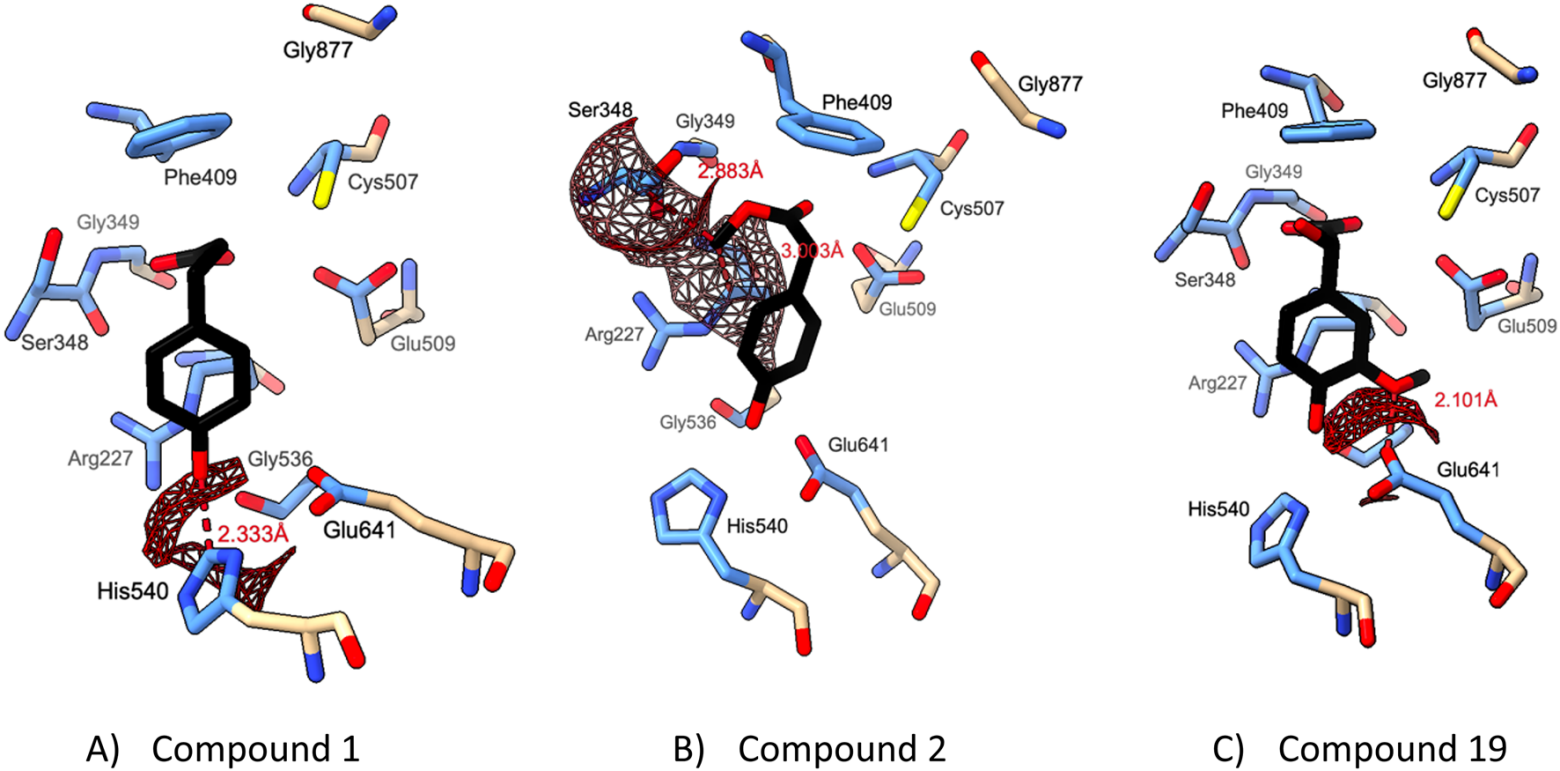
Van der Waals clashes observed for docked compounds 1, 2, and 19 with *C. difficile* enzyme model. Compounds are displayed in black. Van der Waals clashes were identified in ChimeraX using a cut off of 0.6 Å overlap. Distance between clashing atoms labelled in red and depicted by dashed line. Active site residues only are displayed. Atoms within 5 Å of ligand are in blue and the remaining atoms are in beige. Image generated in UCSF ChimeraX.

We propose that the novel compounds characterised here are likely to form hydrogen bonds with Glu641 using their 4′ hydroxyl groups, anchoring them to the active site. Additionally, our modelling suggests that all compounds, except compound 8, are able to form a hydrogen bond with Ser348. We note that the interaction is lost between compound 3 and Cys507, as its single 1′ hydroxyl group is coordinated by Ser348 at a distance of 5.21 Å from the active site cysteine, preventing hydrolysis. In contrast, compounds 1, 9, 17 and 19 are positioned, such that their carboxylate group remains close to the active site cysteine. The highest docking energies observed likely stem from clashes caused by changes to the functional group attached to the 1′ carbon (compound 2), or the additional hydroxyl group on the benzene ring (compound 19), as shown in Fig. 7. Compound 1 was also found to have a clash involving its 4′ hydroxyl (Fig. 7A), which may explain its lack of efficacy in reducing *p*-cresol production when assessed by HPLC–DAD (Fig. 3).

Collectively, the results of our docking experiment correlate well with the experimental data reported in this study. Specifically, of the compounds with the lowest binding energy (compounds 1, 3, 6 and 8), compounds 6 and 8 were found to significantly reduce *p*-cresol production whilst compound 3 was found to give a reduction in *p*-cresol production that was approaching significance (*p* = 0.0528). Compounds 9, 2 and 19 showed significantly higher binding energy, and had the lowest inhibitory activity in our experimental assays.

However, we note that, compound 1 was found to have a binding energy close to that of compound 6, even though it did not inhibit *p*-cresol production. In contrast, compound 2 was found to give a significant reduction in *p*-cresol production despite having a relatively high binding energy, as well as being predicted to have multiple atomic clashes with HpdB (Fig. 7). Compound 17 had a relatively high energy of binding to the enzyme according to our docking procedure, yet it inhibited *p*-cresol production according to our HPLC–DAD assay, and reduced *C. difficile*’s competitiveness in the competition-index assays. This discrepancy between the in silico modelling and experimental characterization of the efficacy of these compounds may be because of differences in uptake kinetics, or compound metabolism.

## Discussion

The interaction between *C. difficile* and the microbiome is vital for CDI and relapse, with a key feature of this relationship being *C. difficile*’s ability to produce *p*-cresol which provides it with a competitive advantage against select bacteria of the microbiome^12^. Unlike other *C. difficile* virulence factors, such as toxin production^32,33^ and sporulation^32^, which differ between clades, utilisation of *p*-HPA for *p*-cresol production is conserved in all five clades of *C. difficile*^17^. Therefore, targeting *p*-cresol production would be equally effective across all strains of *C. difficile* that cause infection. In this study, we have identified several compounds that inhibit *p*-cresol production and *C. difficile*’s ability to compete with a gut dwelling *E. coli* strain. This data demonstrates the viability of reducing *p*-cresol production as a strategy for the selective treatment of *C. difficile* as well as providing a basis for further development of the identified compounds to improve their efficacy and reduce off-target effects.

In a previous study, it was demonstrated that at concentrations of ≥ 2 mg/ml for *C. difficile* 630Δ*erm* and ≥ 1 mg/ml for *E. coli p*-HPA significantly inhibited growth when grown in BHIS media^17^. Therefore, as the putative inhibitors in this study are analogues of *p*-HPA we sought to determine if these compounds had any effects on growth of either species which could suggest they may cause damage to the microbiome. This is particularly important for the development of novel *C. difficile* treatments as reduced infection relapse rates are associated with therapeutics that are microbiome sparing^34^. Whilst four of these compounds: 2, 3, 6 and 8, were inhibitory to *E. coli* at the concentration tested, an equal number either had no effect or promoted *E. coli* growth. Of those that were inhibitory to *E. coli* growth, compounds 2, 6 and 8 were amongst those that inhibited *p*-cresol production (Fig. 3). Additionally, inhibition of *C. difficile* growth was found to occur with three compounds, two of which were only inhibitory in the presence of *p*-HPA also. Therefore, this data promisingly shows that modifications to these compounds could allow for the identification of a compound that is both effective at inhibiting HpdBCA as well as being microbiome sparing.

We have identified several compounds that inhibit *C. difficile p*-cresol production with the standout lead compound, compound 8, (4-Hydroxyphenylacetonitrile), reducing *p*-cresol production by 99.0 ± 0.4% when 630Δ*erm* was grown on its own (Fig. 3) and 61.5 ± 8.4% in the competition-index assay (Fig. 4E). Furthermore, HPLC–DAD analyses from 630∆*erm* showed that compounds 2, 6 and 17 were all found to cause significant reductions in *p*-cresol production, with compound 3 giving a reduction approaching significance also (*p* = 0.0528).

Using in silico analysis, we were able to identify putative interactions between the compounds and HpdB which likely contribute to the inhibition of *p*-cresol production. We observed that the order of docking affinities largely matched the experimental data and is therefore likely to be accurate. Nonetheless, we emphasise that experimental structural validation would be needed to verify this in another study. In contrast, despite their relatively high predicted binding energy compounds 2 and 17 were found to significantly reduce *p*-cresol production. We propose that these discrepancies may be explained by a difference in cellular uptake kinetics for each compound. Accordingly, we speculate that compounds 2 and 17 may be imported into the cell relatively well and therefore reach higher intracellular concentrations than some of the other compounds with higher binding affinities but which did not significantly decrease *p*-cresol production, such as compounds 1 and 9. These compounds’ lack of efficacy could be a result of poor uptake into *C. difficile*, causing relatively low intracellular concentrations insufficient to inhibit *p*-cresol production. Although in the case of compound 1 the lack of efficacy may be due to a possible atomic clash. Further characterisation of compound uptake, and biochemical quantification of enzyme inhibition would be required to verify this.

In the competition-index assays, compounds 1, 3, 6, 8 and 17 were all found to significantly reduce *C. difficile*’s ability to compete with *E. coli*. This was expected with compounds 3, 6 and 8 owing to the reductions in *p*-cresol production identified by HPLC–DAD and the modelling data, which predicted these compounds to have the highest binding affinities. Furthermore, owing to the significant reduction in *p*-cresol production identified by HPLC–DAD this was also expected for compound 17 despite its lower predicted binding affinity. A significant reduction in *C. difficile*’s competitiveness was not expected with compound 1 based on the HPLC–DAD results. A potential reason for this is that during competition-index experiments, the putative inhibitors may be metabolised by *C. difficile* or *E. coli* to generate alternative metabolites, other than *p*-cresol, which reduce the relative fitness of *C. difficile* or *E. coli*. Additionally, conversion to other metabolites may provide an explanation for the lack of significant *p*-cresol reduction in samples analysed from the competition-index assays, with the exception of compound 8. If these compounds are metabolised by *C. difficile* or *E. coli* to other products that cannot bind HpdBCA, then *p*-cresol production will increase over the course of the competition-index experiment as the putative inhibitors are metabolised. As such, compounds may be initially effective at reducing *p*-cresol production, allowing *E. coli* to grow and compete more effectively with *C. difficile*, but over the course of the competition-index assay the concentration of these compounds falls sufficiently so that *p*-cresol production may increase by the end of the co-culture period such that a change in *p*-cresol concentration is not identified.

The efficacy of microbiome sparing treatments and FMT in reducing episodes of infection relapse demonstrate the importance of the restoration of colonisation resistance for the successful treatment of *C. difficile*. Further improvements in *C. difficile* treatment will require therapies to be highly specific against *C. difficile* and to promote the microbiome’s recovery of its lost colonisation resistance. In this work we have identified several promising compounds that demonstrate the potential to fulfil these criteria through the inhibition of *p*-cresol production.

## Supplementary Information

Supplementary Information.

## Data Availability

The datasets used and/or analysed during the current study are available from the corresponding author on reasonable request.

## References

[1] Desai K, Gupta SB, Dubberke ER, Prabhu VS, Browne C, Mast TC (2016). Epidemiological and economic burden of *Clostridium difficile* in the United States: Estimates from a modeling approach. BMC Infect. Dis..

[2] McGlone SM, Bailey RR, Zimmer SM, Popovich MJ, Tian Y, Ufberg P (2012). The economic burden of *Clostridium difficile*. Clin Microbiol. Infect..

[3] Bartlett JG, Gerding DN (2008). Clinical recognition and diagnosis of *Clostridium difficile* infection. Clin. Infect. Dis..

[4] Brown KA, Langford B, Schwartz KL, Diong C, Garber G, Daneman N (2020). Antibiotic prescribing choices and their comparative *C. difficile* infection risks: A longitudinal case-cohort study. Clin. Infect. Dis..

[5] Rousseau C, Poilane I, De Pontual L, Maherault AC, Le Monnier A, Collignon A (2012). *Clostridium difficile* carriage in healthy infants in the community: A potential reservoir for pathogenic strains. Clin. Infect. Dis..

[6] Marsh JW, Arora R, Schlackman JL, Shutt KA, Curry SR, Harrison LH (2012). Association of relapse of *Clostridium difficile* disease with BI/NAP1/027. J. Clin. Microbiol..

[7] Vardakas KZ, Polyzos KA, Patouni K, Rafailidis PI, Samonis G, Falagas ME (2012). Treatment failure and recurrence of *Clostridium difficile* infection following treatment with vancomycin or metronidazole: A systematic review of the evidence. Int. J. Antimicrob. Agents.

[8] Moayyedi P, Yuan Y, Baharith H, Ford AC (2017). Faecal microbiota transplantation for *Clostridium difficile*-associated diarrhoea: A systematic review of randomised controlled trials. Med. J. Aust..

[9] Hocquart M, Lagier JC, Cassir N, Saidani N, Eldin C, Kerbaj J (2018). Early fecal microbiota transplantation improves survival in severe *Clostridium difficile* infections. Clin. Infect. Dis..

[10] DeFilipp Z, Bloom PP, Torres Soto M, Mansour MK, Sater MRA, Huntley MH (2019). Drug-resistant *E. coli* bacteremia transmitted by fecal microbiota transplant. N. Engl. J. Med..

[11] Zellmer C, Sater MRA, Huntley MH, Osman M, Olesen SW, Ramakrishna B (2021). Shiga toxin-producing *Escherichia coli* transmission via fecal microbiota transplant. Clin. Infect. Dis..

[12] Passmore IJ, Letertre MPM, Preston MD, Bianconi I, Harrison MA, Nasher F (2018). *Para*-cresol production by *Clostridium difficile* affects microbial diversity and membrane integrity of Gram-negative bacteria. PLoS Pathog..

[13] Saito Y, Sato T, Nomoto K, Tsuji H (2018). Identification of phenol- and p-cresol-producing intestinal bacteria by using media supplemented with tyrosine and its metabolites. FEMS Microbiol. Ecol..

[14] Selmer T, Andrei PI (2001). p-hydroxyphenylacetate decarboxylase from *Clostridium difficile*. A novel glycyl radical enzyme catalysing the formation of p-cresol. Eur. J. Biochem..

[15] Dawson LF, Donahue EH, Cartman ST, Barton RH, Bundy J, McNerney R (2011). The analysis of *para*-cresol production and tolerance in *Clostridium difficile* 027 and 012 strains. BMC Microbiol..

[16] Harrison MA, Faulds-Pain A, Kaur H, Dupuy B, Henriques AO, Martin-Verstraete I (2020). *Clostridioides difficile para*-Cresol production is induced by the precursor *para*-hydroxyphenylacetate. J. Bacteriol..

[17] Harrison MA, Kaur H, Wren BW, Dawson LF (2021). Production of *p*-cresol by decarboxylation of *p*-HPA by all five lineages of *Clostridioides difficile* provides a growth advantage. Front. Cell. Infect. Microbiol..

[18] Martins BM, Blaser M, Feliks M, Ullmann GM, Buckel W, Selmer T (2011). Structural basis for a Kolbe-type decarboxylation catalyzed by a glycyl radical enzyme. J. Am. Chem. Soc..

[19] Yu L, Blaser M, Andrei PI, Pierik AJ, Selmer T (2006). 4-Hydroxyphenylacetate decarboxylases: Properties of a novel subclass of glycyl radical enzyme systems. Biochemistry.

[20] Andrei PI, Pierik AJ, Zauner S, Andrei-Selmer LC, Selmer T (2004). Subunit composition of the glycyl radical enzyme p-hydroxyphenylacetate decarboxylase. Eur. J. Biochem..

[21] Hussain HA, Roberts AP, Mullany P (2005). Generation of an erythromycin-sensitive derivative of *Clostridium difficile* strain 630 (630Deltaerm) and demonstration that the conjugative transposon Tn916DeltaE enters the genome of this strain at multiple sites. J. Med. Microbiol..

[22] Cartman ST, Minton NP (2010). A mariner-based transposon system for in vivo random mutagenesis of *Clostridium difficile*. Appl. Environ. Microbiol..

[23] The UniProt Consortium (2017). UniProt: The universal protein knowledgebase. Nucleic Acids Res..

[24] Waterhouse A, Bertoni M, Bienert S, Studer G, Tauriello G, Gumienny R (2018). SWISS-MODEL: Homology modelling of protein structures and complexes. Nucleic Acids Res..

[25] Hinsen K (2000). The molecular modeling toolkit: A new approach to molecular simulations. J. Comput. Chem..

[26] Kim S, Chen J, Cheng T, Gindulyte A, He J, He S (2020). PubChem in 2021: New data content and improved web interfaces. Nucleic Acids Res..

[27] Pettersen EF, Goddard TD, Huang CC, Couch GS, Greenblatt DM, Meng EC (2004). UCSF Chimera—A visualization system for exploratory research and analysis. J. Comput. Chem..

[28] Morris GM, Huey R, Lindstrom W, Sanner MF, Belew RK, Goodsell DS (2009). AutoDock4 and AutoDockTools4: Automated docking with selective receptor flexibility. J. Comput. Chem..

[29] Eberhardt J, Santos-Martins D, Tillack AF, Forli S (2021). AutoDock Vina 1.2.0: New docking methods, expanded force field, and python bindings. J. Chem. Inf. Model..

[30] Feliks M, Martins BM, Ullmann GM (2013). Catalytic mechanism of the glycyl radical enzyme 4-hydroxyphenylacetate decarboxylase from continuum electrostatic and QC/MM calculations. J. Am. Chem. Soc..

[31] Frey M, Rothe M, Wagner AF, Knappe J (1994). Adenosylmethionine-dependent synthesis of the glycyl radical in pyruvate formate-lyase by abstraction of the glycine C-2 pro-S hydrogen atom. Studies of [2H]glycine-substituted enzyme and peptides homologous to the glycine 734 site. J. Biol. Chem..

[32] Akerlund T, Persson I, Unemo M, Norén T, Svenungsson B, Wullt M (2008). Increased sporulation rate of epidemic *Clostridium difficile* type 027/NAP1. J. Clin. Microbiol..

[33] Merrigan M, Venugopal A, Mallozzi M, Roxas B, Viswanathan VK, Johnson S (2010). Human hypervirulent *Clostridium difficile* strains exhibit increased sporulation as well as robust toxin production. J. Bacteriol..

[34] Cornely OA, Miller MA, Louie TJ, Crook DW, Gorbach SL (2012). Treatment of first recurrence of *Clostridium difficile* infection: Fidaxomicin versus vancomycin. Clin. Infect. Dis..

