# Supplementary material for: Identification of novel p-cresol inhibitors that reduce Clostridioides difficile’s ability to compete with species of the gut microbiome
Source: Sci Rep. 2023 Jun 11;13:9492. doi: 10.1038/s41598-023-32656-8 (PMC10258198; doi:10.1038/s41598-023-32656-8)
Supplement: Supplementary file 1 — Supplementary Information. [file 41598_2023_32656_MOESM1_ESM.docx]

Identification of novel *p*-cresol inhibitors that reduce *Clostridioides difficile*’s ability to compete with species of the gut microbiome

Mark A. Harrison^1^, Rebecca J. Farthing^2^, Nyasha Allen^1^, Lucy M. Ahern^1^, Kristian Birchall^4^, Michael Bond^4^, Harparkash Kaur^3^, Brendan W. Wren^1^, Julien R.C Bergeron^2^ and Lisa F. Dawson^1^*

Supplementary figures and tables


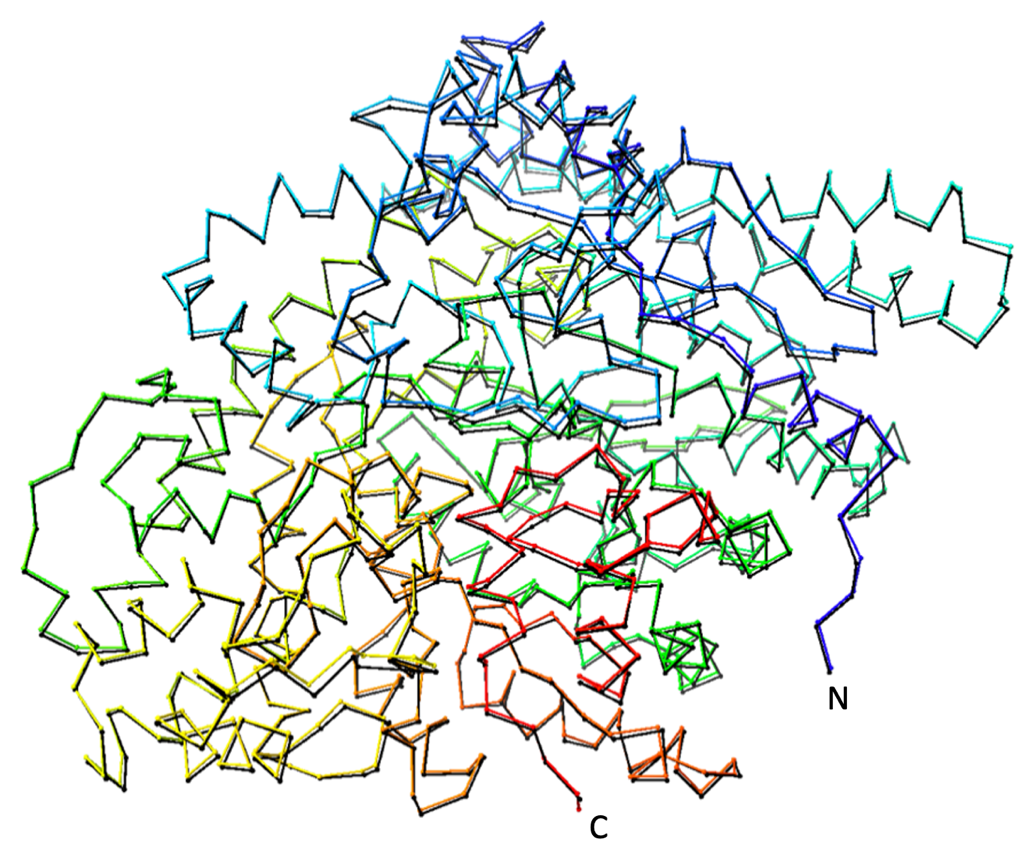


**Supplementary figure 1. Structural alignment of *C. difficile* HpdB homology model with crystal structure of *C. scatologenes* HpdB (PDB ID: 2YAJA) (25).** *C. difficile* is displayed in black and *C. scatologenes* in rainbow (from N to C terminus). The carbon trace only is displayed. Figure generated in UCSF ChimeraX.


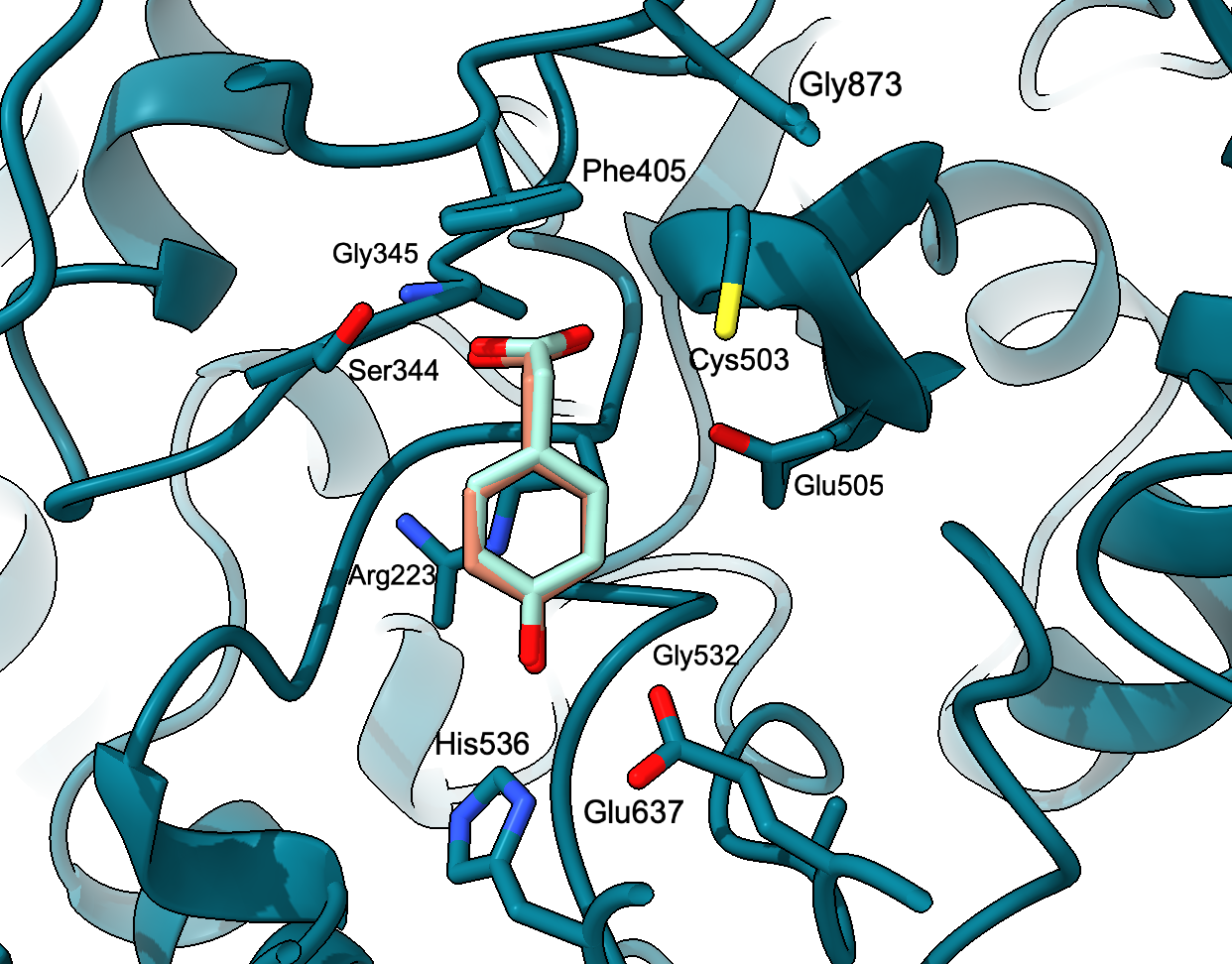


**Supplementary Figure 2. Docking of the substrate *p*-hydroxyphenylacetate leads to the same binding mode as observed in the experimentally determined structure**. The *C. scatologenes* HpdB crystal structure (PDB ID: 2YAJA) is shown in teal, in cartoon representation (25). *p*-HPA from the experimentally determined complex structure is shown in orange, and the lowest-energy solution from the docking procedure is in mint green. Side chains of the active site residues for HpdB are displayed as sticks, and the residue numbers correspond to the *C. difficile* sequence. Figure generated in UCSF ChimeraX.

| *para*-hydroxyphenylacetic acid  (*p-*HPA) | 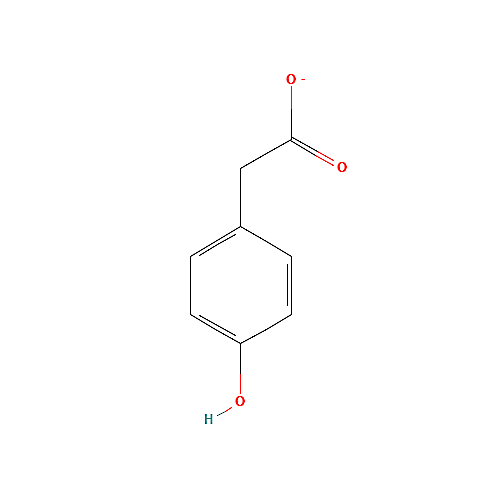 | 4-Hydroxyphenylacetonitrile  (Compound 8) | 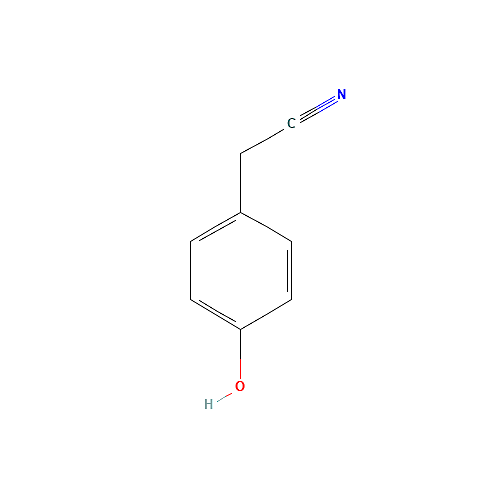 | |
| --- | --- | --- | --- | --- |
| 3-(4-Hydroxyphenyl)propionic acid  (Compound 1) | *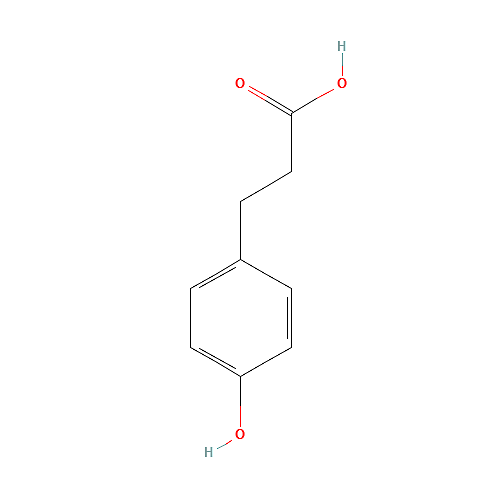* | 3,4-Dihydroxyphenylacetic acid  (Compound 9) | 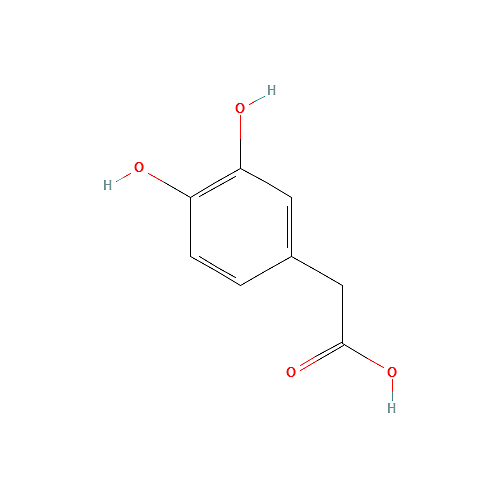 | |
| Methyl 4-hydroxyphenylacetate  (Compound 2) | *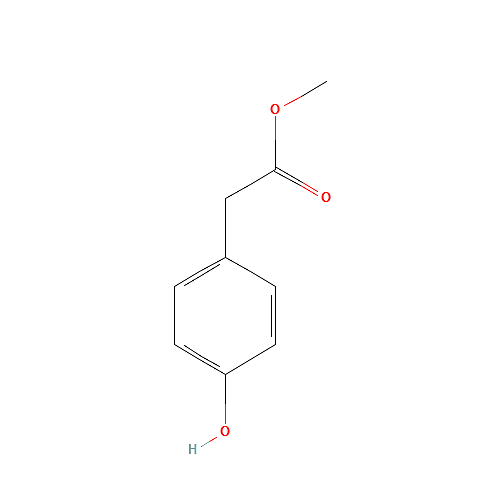* | 4-Hydroxybenzoic acid  (Compound 17) | 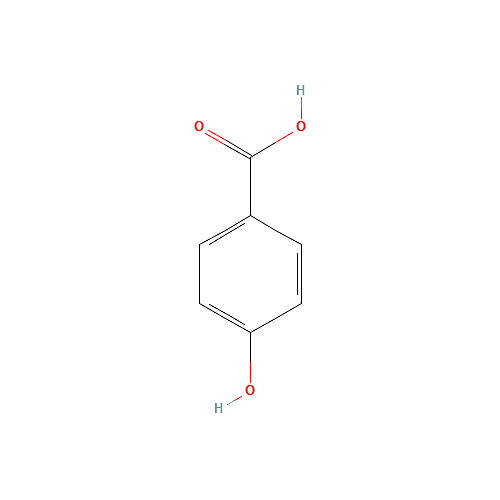 | |
| 2-(4-Hydroxyphenyl)ethanol  (Compound 3) | 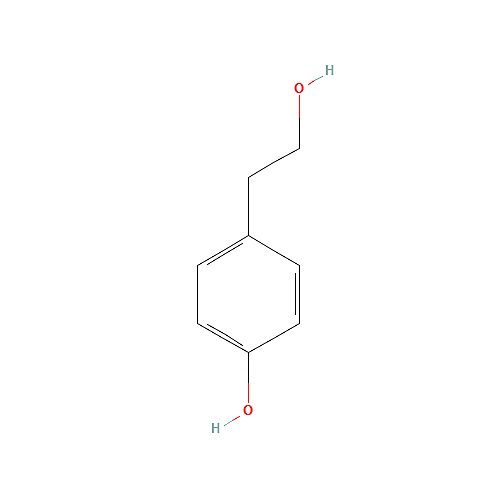 | DL-4-Hydroxy-3-methoxymandelic acid  (Compound 19) | 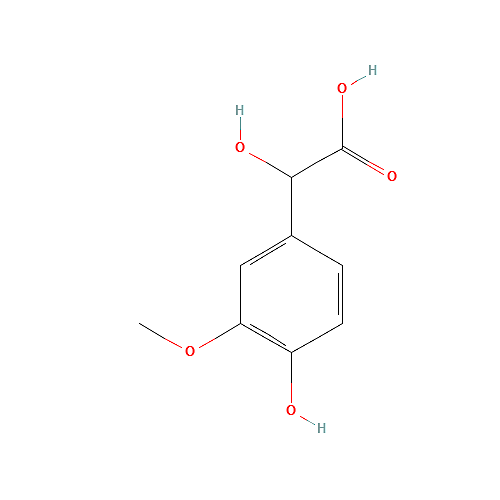 |  |
| 4-Hydroxyphenylacetamide  (Compound 6) | 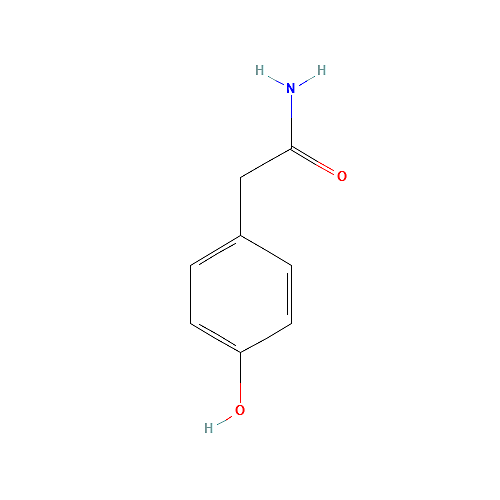 | (2RS)-2-(4-Hydroxyphenyl)propionic acid | 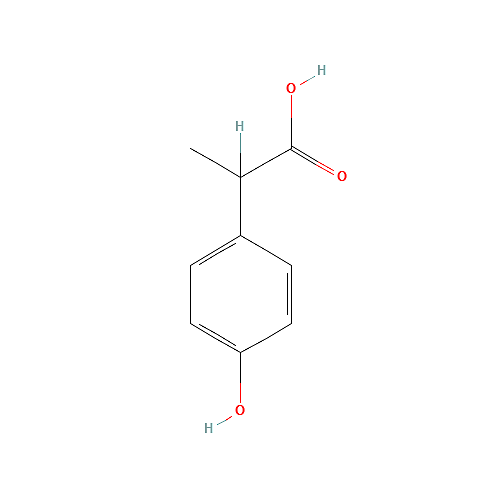 |  |
| 2-(4-Hydroxyphenyl)-2-methylpropanoic acid | 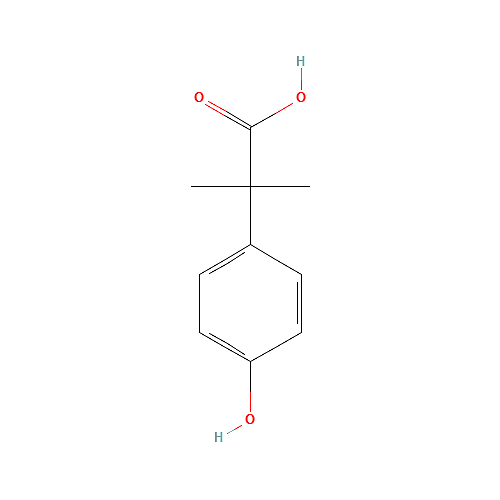 | 2-(3-amino-4-hydroxyphenyl)acetic acid | 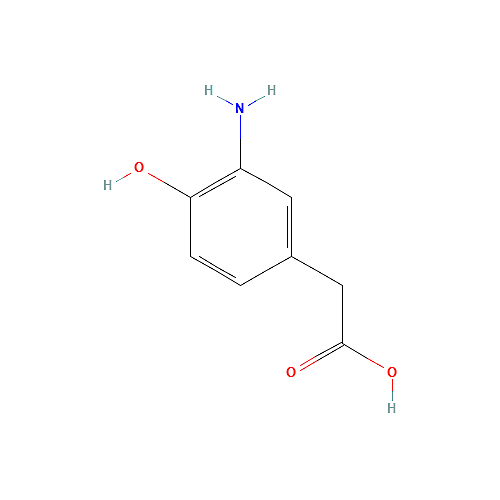 |  |
| 4-Bromophenylacetic acid | 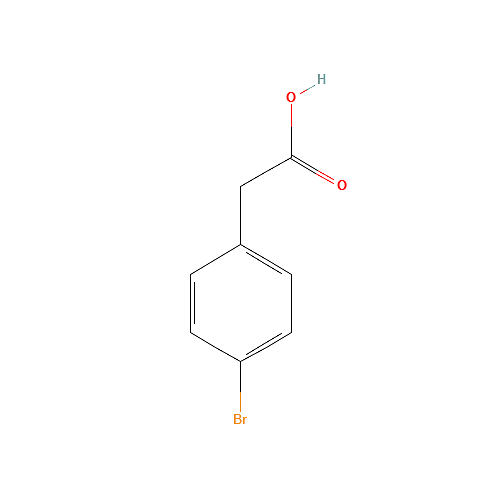 | 3-Bromo-4-hydroxyphenylacetic acid | 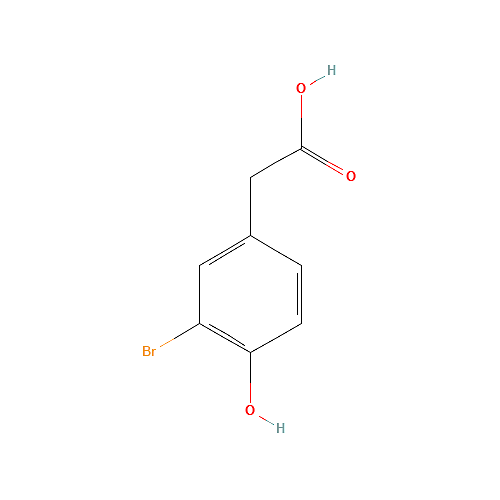 |  |
| 4-(2-Methoxyethyl)phenol | 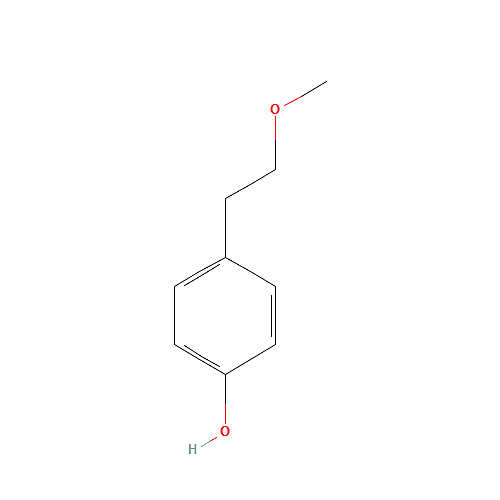 | 3-Chloro-4-hydroxyphenylacetic acid | 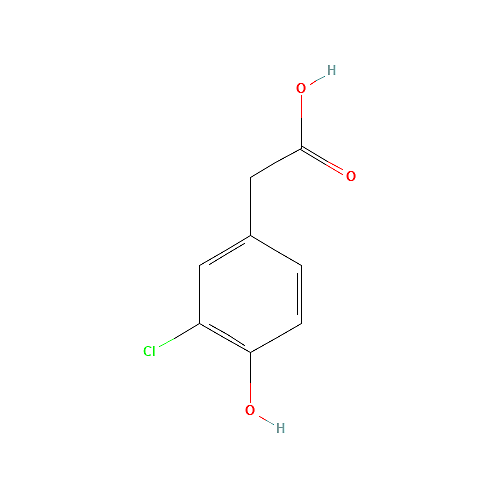 |  |
| 4-aminophenylacetic acid | 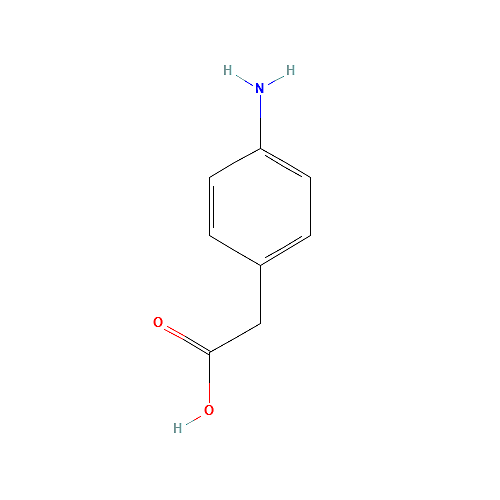 | 4-Hydroxy-3-methoxyphenylacetic acid | 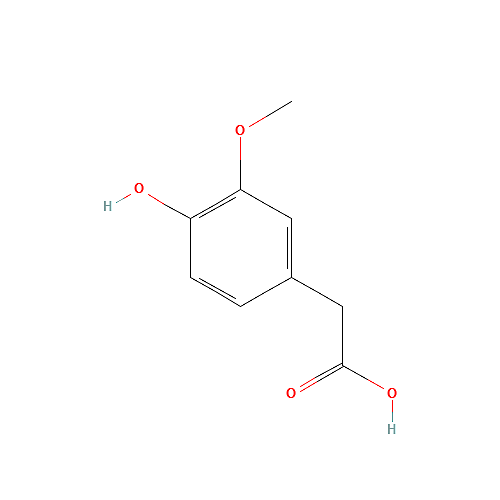 |  |
| 4-Tolylacetic acid | 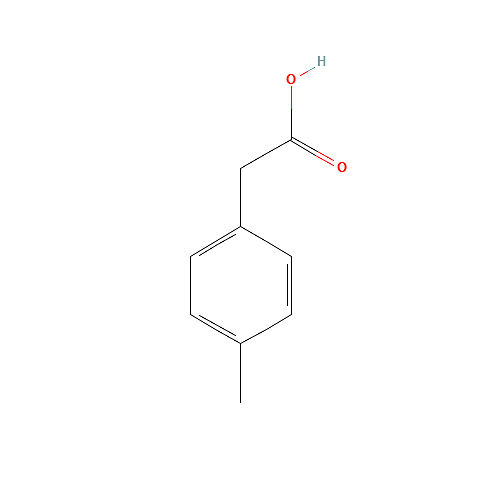 | 2-Fluoro-4-hydroxyphenylacetic acid | 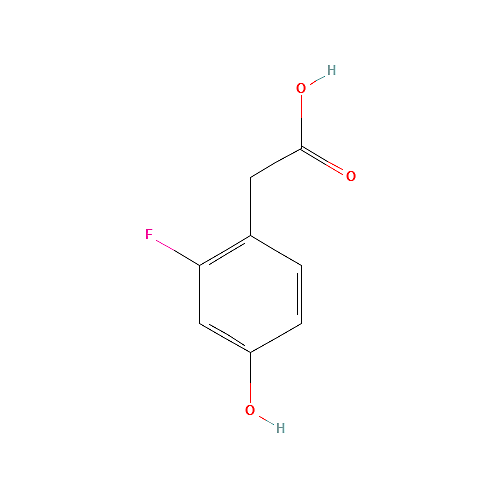 |  |
| 2-Hydroxy-2-(4-hydroxyphenyl)acetic acid | 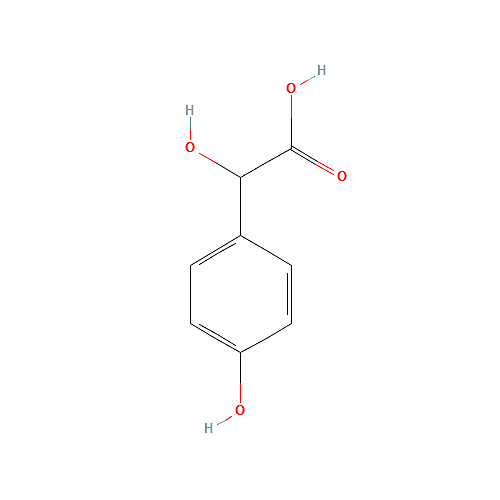 | 2-Amino-2-(4-hydroxyphenyl)acetic acid | 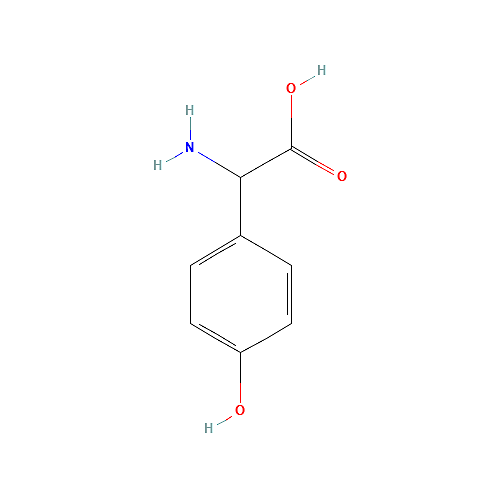 |  |
| 1-(4-Hydroxyphenyl)propan-2-one | 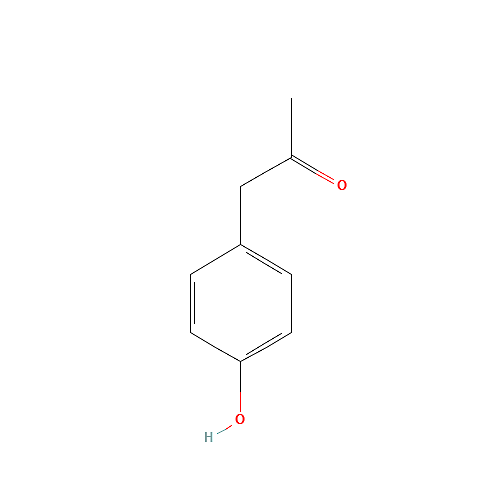 | 3-Hydroxyphenylacetic acid | 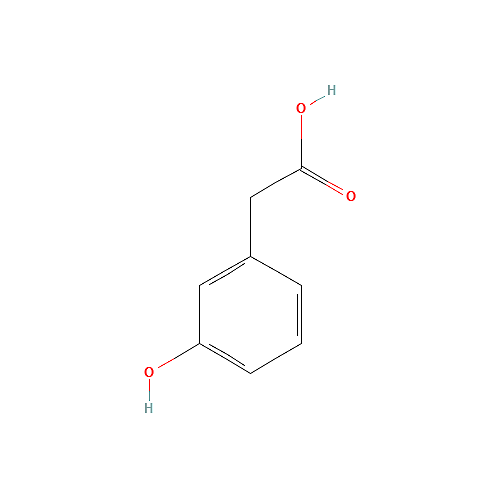 |  |
| 2-(4-Hydroxycyclohexyl)acetic acid | 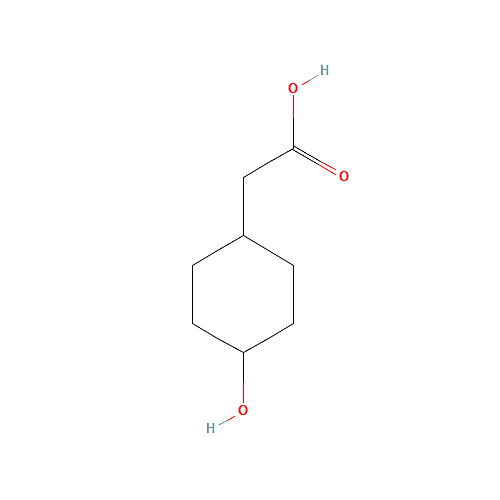 | 3-Methyl-4-hydroxyphenylacetic acid | 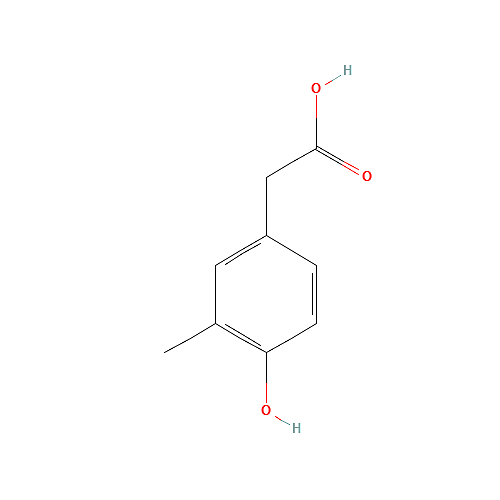 |  |
| 2-(4-aminopiperidin-1-yl)acetic acid | 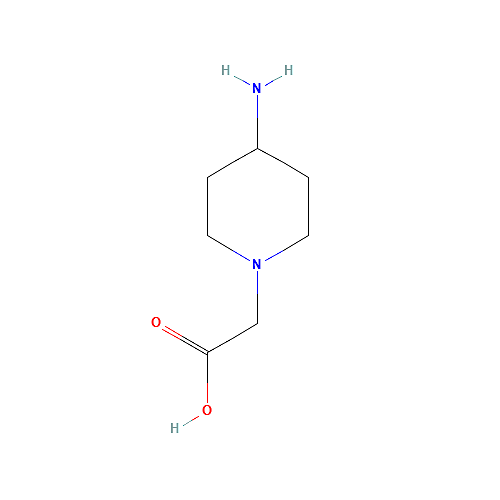 | 3-Fluoro-4-hydroxyphenylacetic acid | 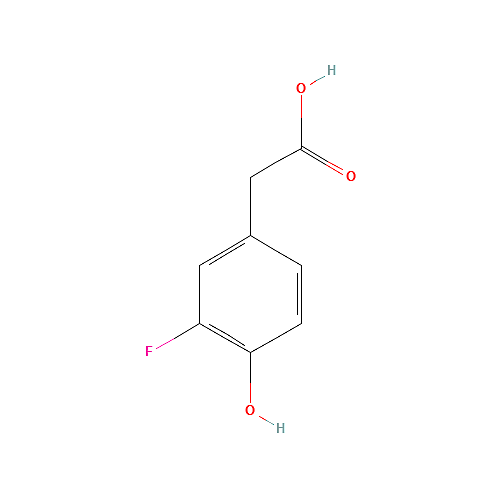 |  |
| 4-Methoxyphenylacetic acid | 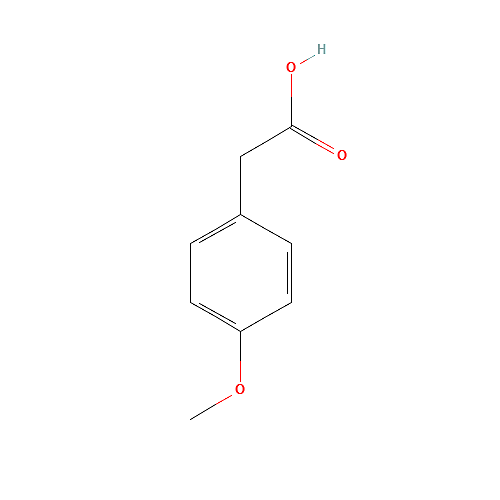 | Phenylacetic acid | 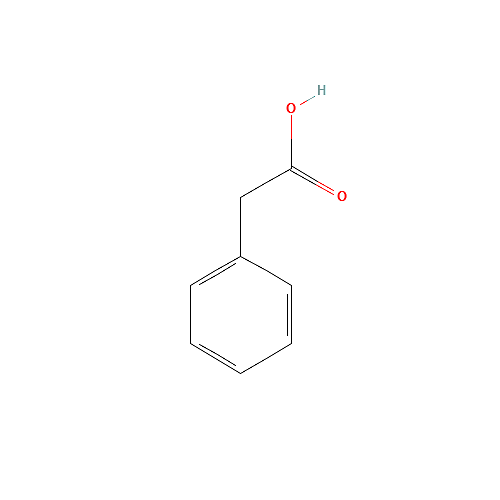 |  |

**Supplementary table 1.** Structures of *p*-HPA and the compounds identified through substructure and similarity searching of catalogues of commercially available compounds as analogues of *p*-HPA. Structural images are from the PubChem database (1).

References

1. Kim S, Chen J, Cheng T, Gindulyte A, He J, He S, et al. PubChem in 2021: new data content and improved web interfaces. Nucleic Acids Research. 2020;49(D1):D1388-D95.
